# Kinetic and Thermodynamic Investigation into the Formation of Diol-Boric Acid Complexes Using NMR and Computational Analysis

**DOI:** 10.1021/acsomega.5c07314

**Published:** 2025-11-17

**Authors:** Brandy L. Davidson, Elaina Manyin, Nathan E. Morris, Isaiah Sumner, Brycelyn M. Boardman, Gretchen M. Peters

**Affiliations:** Department of Chemistry and Biochemistry, 3745 James Madison University, Harrisonburg, Virginia 22807, United States

## Abstract

Boric acid (BA) readily forms reversible covalent B–O bonds with diols, yielding complexes with broad implications for drug delivery, materials science, and polymer chemistry. While this chemistry is well-established in aqueous environments, little is known about the formation of neutral boron complexes with BA in organic solvents. Here, we describe a spectroscopic and theoretical study of the complexation of diols with BA in DMSO. NMR spectroscopy was used to evaluate the formation of complexes for three structurally distinct diols: ethylene glycol (EG), 1,3-propanediol (PD), and 1,4-butanediol (BD). Results indicate that with BA, PD forms the most closed B–O complex, while EG forms a mixture of open and closed B–O species, and BD forms only small amounts of the open diol-BA complex. These findings were supported computationally for both PD and EG, where the Δ*G* between the open and closed B–O complexes was −6.86 and −1.45 kcal/mol, respectively. For BD-BA, the open and closed complexes were found to be similar in energy, which was not observed experimentally. We hypothesize that this is the result of a kinetic sink. The rates of formation and the impact of water were also evaluated using NMR techniques. Kinetic analysis determined that all reactions were first order with respect to diol, with rate constants of 0.057 min^–1^, 0.031 min^–1^, and 0.124 min^–1^ for EG-BA, PD-BA, and BD-BA, respectively. In combination, our results indicate that the closed 1,3-diol-BA complexes are more thermodynamically stable, while the 1,2-diol-BA complex is kinetically favored and dynamic. Additionally, theoretical methods were used to probe the impact of stabilizing interactions of polyol-BA complexes with glycerol (Glyc). Namely, an intramolecular hydrogen bond within 1,2-Glyc-BA was found to significantly stabilize this complex and lead to similar relative energies for both isomers (ΔΔ*G* of −0.57 kcal/mol). This result corroborated our previously reported experimental data, which showed an ∼50:50 mixture of the 1,2- and 1,3-Glyc-BA isomers.

## Graphical abstract

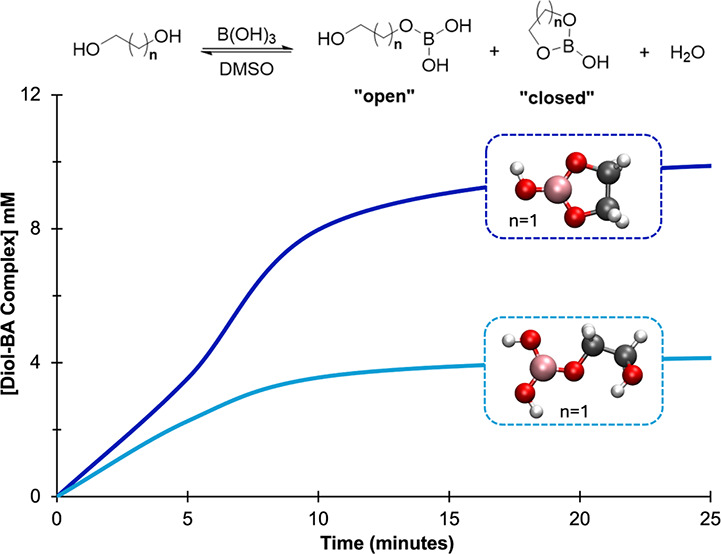

## Introduction

Diols are ubiquitous and highly valuable compounds. They are widely used as coolants and plasticizers, and in the tobacco,^1^ textile, and cosmetic industries.^2^ Diols are industrial precursors to polyesters^3^ and polyurethanes^4^ and are common building blocks for synthesis.^5^ Because of their ubiquity, expanding our understanding of reactions involving diol compounds is of great value. One reaction that is of particular interest is the complexation of diols with boric acid (BA). In the presence of BA, diols readily form reversible, covalent B–O bonds, yielding borate ester complexes. This chemistry has been exploited in a number of different areas from drug delivery,^6,7^ to self-healing materials,^8–11^ and hydrogels.^12–17^ Generally, diol-BA complexation has been explored in aqueous basic conditions through the formation of tetravalent borate ester cross-links.^18–22^ However, in recent years, it has been shown that BA can also form complexes in organic media, such as DMSO.^10,23–27^

Our previous work with polyol-based plasticizers has utilized diol-BA chemistry and highlights the complexity of these systems.^23,28^ In an effort to modulate the properties of bioplastics, we introduced BA to polyols of varying length. In this work, we found that glycerol (Glyc) formed two distinct neutral boron complexes in DMSO (Figure 1).^23^ These isomeric complexes, 1,3-Glyc-BA and 1,2-Glyc-BA, form a ∼50:50 mixture. With tetrol erythritol (Ery), we also observe the formation of 1,3- and 1,2-Ery-BA complexes, but the ratio of these species is notably different. In this case, the 1,3-Ery-BA complex is formed at ∼85%, while the 1,2-Ery-BA complex makes up only ∼15%. Additionally, little to no 2,3-Ery-BA is observed (structure not shown).^28^ Extensive studies into the impacts of the structure on diol-BA complexation in organic solvents remain largely unexplored. Furthermore, little is known about the kinetics and thermodynamics of this reaction under these conditions.

**Figure 1:**
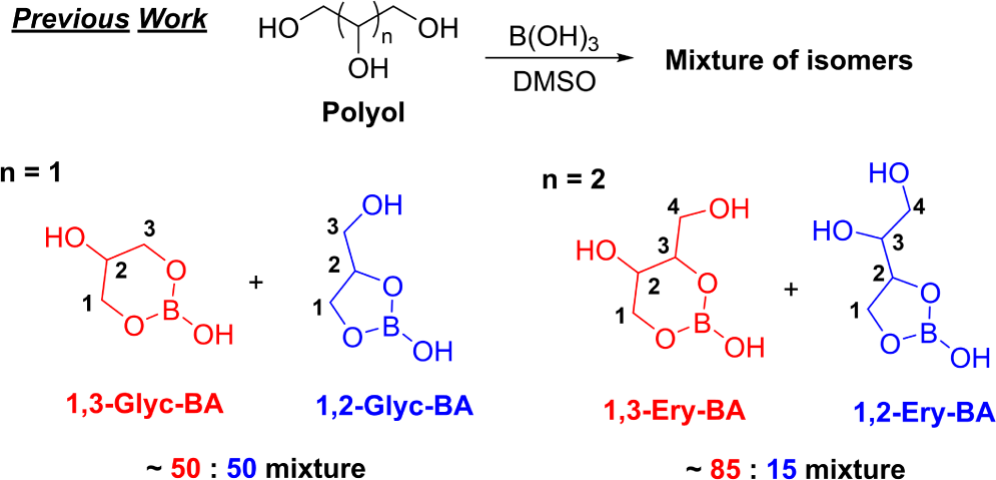
Previous studies of Glyc-BA systems indicate the formation of a nearly 50:50 mixture of 1,3- and 1,2-Glyc-BA complexes. Extending the length of the polyol resulted in a clear preference for the 1,3-isomer, as seen with the Ery B–O ester formation.

Herein, we detail a spectroscopic and computational investigation of the kinetics and thermodynamics of the formation of neutral diol-BA complexes in DMSO. Three diols, ethylene glycol (EG), 1,3-propanediol (PD), and 1,4-butanediol (BD), were chosen to probe the impact of diol chain length on the formation of neutral boron complexes. Spectroscopically, the formation of cyclic complexes (closed) was observed for PD-BA and EG-BA, with PD-BA forming a significantly higher concentration of the closed complex than EG-BA (Figure 2 and SI-Figure S0). EG-BA also forms an open complex in which BA binds to only one hydroxyl unit of the diol. The 1,4-isomer (BD) forms exclusively open complexes with BA. Computational analysis of the open versus closed forms of these species confirmed the spectroscopic results, indicating that the closed PD-BA complex is more thermodynamically favored than the closed forms of EG-BA or BD-BA. Kinetic analysis of the NMR data indicates that all complexation reactions are first-order with respect to diol and zero-order with respect to BA. Additional computational studies with Glyc-BA systems highlighted the importance of both ring stability and secondary interactions on the energetics of polyol-BA systems.

**Figure 2:**
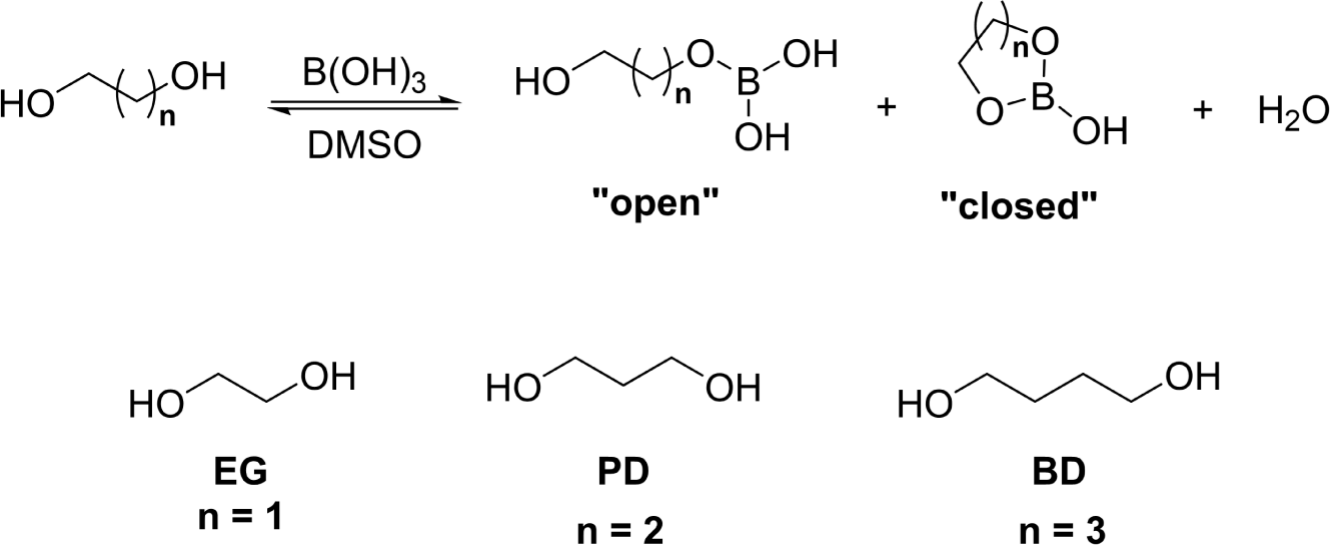
General reaction scheme for the formation of open and closed diol-BA complexes. Structures of diols EG, PD, and BD are also shown, highlighting the increase in the length of the alkyl units. Full structural depictions of open and closed complexes for individuals diols can be found in SI-Figure S0.

## Results and Discussion

The formation of neutral boron complexes yields water as a byproduct. Therefore, to truly understand the impact of chain length on diol-BA complex formation, a detailed investigation of the impact of water on conversion to product was conducted. An NMR titration with increasing water percentages (∼0.5–6%) was performed on EG, PD, and BD (50 mM) with 1 equiv of BA in *d*_6_-DMSO (SI-Figure S1–S3). All samples were allowed to reach equilibrium before the percent complexation of each reaction was determined. When the water percentage increased from ∼0.5% to ∼1.5%, EG-BA and BD-BA complexes showed an ∼60% decrease in product conversion, while the formation of PD-BA complexes decreased by ∼40%. As anticipated, additional increases in the water percentage further decreased the formation of products for all complexes. Based on these findings, all further experiments, unless otherwise noted, were conducted at consistent water content (2% ± 0.12) in an attempt to remove the impact of water on the kinetics and thermodynamics of these systems.

To determine the relative conversion and concentrations of open and closed complexes at equilibrium, NMR experiments were performed at 50 mM BA and 50 mM diol (EG, PD, or BD) in *d*_6_-DMSO. The time to reach equilibrium was determined by monitoring the NMR spectra as a function of time. The diol-containing sample was frozen prior to BA addition and allowed to thaw in the magnet prior to data collection. NMR spectra were collected every minute for the first hour and then every hour for as long as 7 days (or until no changes were observed in the spectrum). From these data, we can see that at equilibrium, 1,3-PD forms the highest concentration of B–O complexes (37.3 mM closed, 0.78 mM open) with a percent conversion from free diol to neutral boron complexes of 76% (Figure 3). EG-BA (10.6 mM closed, 4.2 mM open) and BD-BA complexes (4.4 mM open) are formed in significantly lower concentrations, resulting in a percent complexation to neutral boron complexes of 30% and 9%, respectively. Interestingly, we also observed a notable difference in the stability of these complexes when they are exposed to water. As previously stated, EG-BA shows ∼60% decrease in total complexation (combined open and closed) when the water percentage is increased from ∼0.5% to ∼1.5%. However, if we look at the individual complexes, the open form of EG-BA decreases by 26%, while the closed EG-BA complex displays a 72% decrease (SI-Figure 3). As previously mentioned, in this same water percentage range, PD-BA complexation (closed) decreases by only ∼40%. This would suggest that the closed EG-BA is significantly more susceptible to water than the closed PD-BA and indicates that the cyclic ester of the EG-BA complex is more dynamic than the closed PD-BA. These findings, in conjunction with percent conversions at equilibrium, are consistent with the PD-BA complex being more thermodynamically stable than EG-BA or BD-BA.

**Figure 3:**
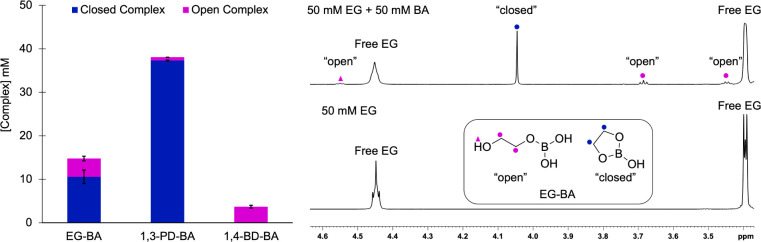
(Left) Plot of the [complex] formed versus diol. Closed complexes are shown in blue, and open complexes are shown in fuchsia. (Right) ^1^H NMR spectra of 50 mM EG and 50 mM EG + 50 mM BA in *d*_6_-DMSO. Resonances for the open (fuchsia) and closed (blue) species are denoted. Spectra of PD and BD alone and with 1 equiv of BA can be found in SI Figures S4 and S5.

To further investigate this finding, we turned to computational analysis to elucidate the difference in cyclic product formation and the impact of linker length between hydroxyl units on open versus closed complexation in these systems. The difference in Gibb’s free energy between open and closed complexes was calculated by subtracting the open structure from the closed structure plus a free water molecule needed to balance the reaction $\left(\Delta G=(G_{\mathrm{closed}}+G_{H_{2}O})-G_{\mathrm{open}}\right)$ . However, we assume that the free water molecule does not interact directly with the closed complex. Negative values of Δ*G* indicate that the closed complex is favored over the open complex. The Δ*G* values for EG-BA, PD-BA, and BD-BA were −1.45 kcal/mol, −6.86 kcal/mol, and −0.82 kcal/mol, respectively (Figure 4).

**Figure 4:**
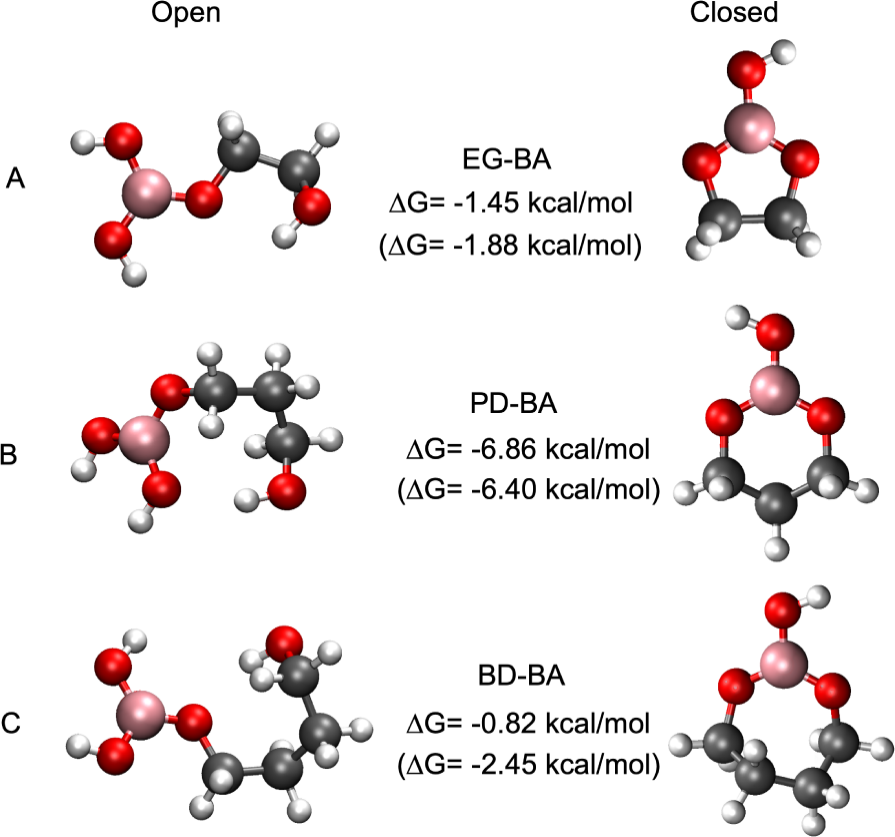
Structural representations and the Gibb’s free energy differences between the open and closed forms of EG-BA, PD-BA, and BD-BA. Only the lowest energy rotamers are shown. The values outside the parentheses are the Δ*G*s when all rotamers are considered, and the values in parentheses are the Δ*Gs* when only the lowest energy rotamers are considered.

Finding all of the rotational conformers for the open structures was important for determining the Δ*G* values, especially for longer-chain diols. As outlined in the Computational Methods section, we used the MSTor^29^ program to determine the total number of unique, stable rotational conformers for each diol. We then used their electronic energies to calculate their Boltzmann distribution (excluding mirror images):

1 ρi=exp(−EikBT)Z

where ρ_*i*_ is the population of the *i*th rotamer, *E_i_* is the electronic energy of the *i*th rotamer, *k*_B_ is Boltzmann’s constant, *T* is the temperature, and Z=∑j=1Nexp(−EjkBT) , where the sum is over all rotamers. The results are shown in Table 1. Most of the low-energy open geometries have a “ring-like” structure similar to the lowest-energy rotamers shown in Figure 4 (SI-Figures S13–S15).

**Table 1:** Total Number of Unique Rotational Isomers and the Boltzmann Distribution Population by Percentile for Each Diol^a^

**Complex**	**Total**	**90%**	**95%**	**99%**
EG-BA	13	6	8	12
PD-BA	38	5	15	30
BD-BA	115	64	79	98

a The distributions were calculated using electronic energies and assuming room temperature (298.15 K).

It is unsurprising that longer-chain diols have more unique structures. However, significantly more conformers make up 90% of the total BD-BA population distribution than EG-BA and PD-BA (64 vs 6 and 5), indicating that it is crucial to consider all possible rotamers for BD-BA (Table 1). We note that there are also two unique closed BD-BA conformations. This point is further supported by noting the significant difference in the Δ*G* calculation when all rotamers were not considered and only the lowest-energy rotamer was taken into account (Figure 4).

These computational results for EG-BA and PD-BA are in good agreement with the spectroscopic data. These findings suggest that the closed PD-BA complex is more thermodynamically favored than the closed forms of EG-BA or BD-BA. However, the Δ*G* of BD-BA indicates that nearly equal amounts of open and closed complexes should be observed, which does not correlate with the spectroscopic data. This may indicate that the open BD-BA complex is kinetically trapped and that in the spectroscopic experiments, thermodynamic equilibrium has not been achieved.

With insights into the thermodynamics of these systems, we next turned our attention to the kinetics of the B–O complex formation. As mentioned previously, diol-containing samples were frozen prior to BA addition and allowed to thaw before data was acquired. Spectra were recorded as a function of time and concentrations of each species were determined (SI-Figures S6–S8). As shown in Figure 5, there is a notable and rapid decrease in diol concentration and an increase in B–O complex formation upon BA addition. Over time, an apparent equilibrium is achieved in all systems. The EG-BA and BD-BA complexes reach equilibrium in ∼20–30 min, whereas PD-BA takes ∼72 h to reach equilibrium (Figure 5 and SI-Figure S9).

**Figure 5:**
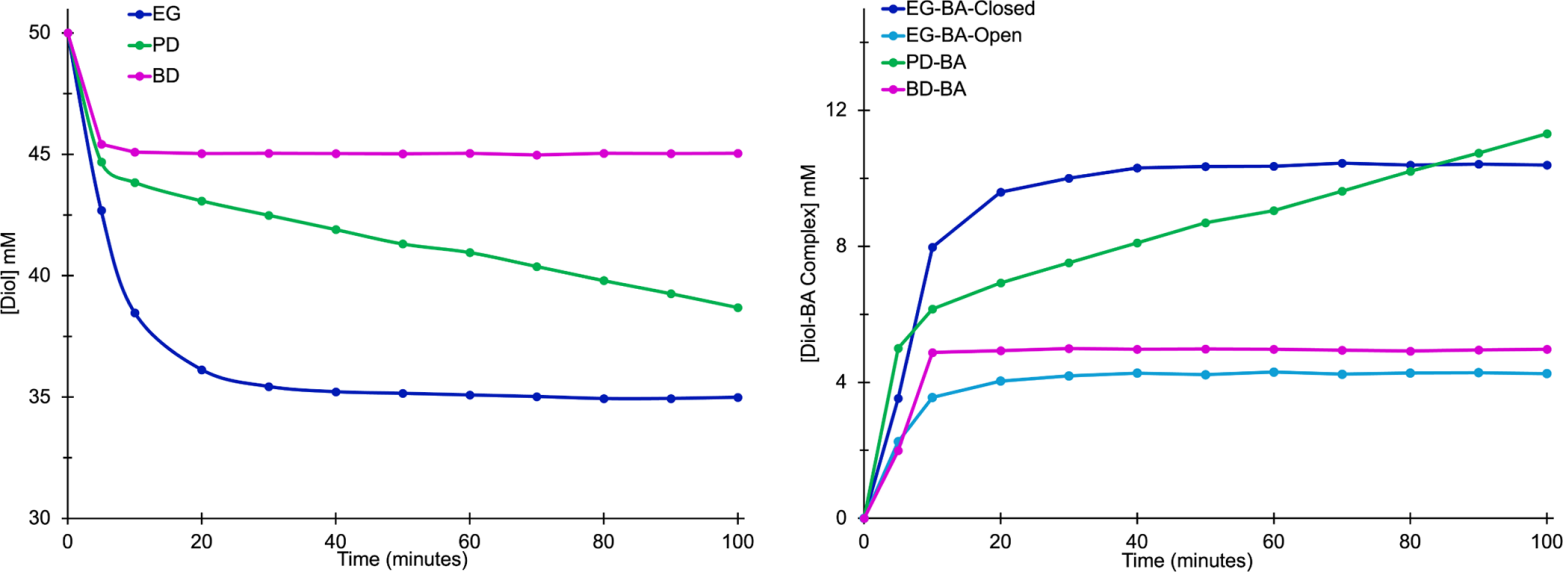
Plots used for kinetic analysis: (left) decreasing [diol] versus time for EG (blue), PD (green), and BD (fuchsia). (Right) Increasing [diol-BA] versus time for closed EG-BA (dark blue), open EG-BA (light blue), PD-BA (green), and BD-BA (fuchsia).

To further explore the kinetics of each of these reactions, three sets of concentrations of diol and BA were used (Table 2, SI-Figures S10 and S11). Experiments were run as described above, and NMR spectra were collected every minute for 100 min. Comparison of the initial rates at different reactant concentrations indicated that all of the reactions were first order with respect to diol as the initial rate doubled when the diol concentration was doubled (∼47.35 mM/min to ∼96.90 mM/min for EG-BA; ∼42.29 mM/min to ∼89.75 mM/min for PD-BA; ∼48.34 mM/min to ∼93.23 mM/min for BD-BA). In contrast, doubling the concentration of BA from 25 mM to 50 mM had little to no impact on the overall rate of the reaction. This indicates that the B–O complexation reaction is zero order with respect to BA.

**Table 2:** Values for Diol and BA Concentration with the Resulting Initial Rates for EG-BA, PD-BA, and BD-BA^a^

		Initial Rate (mM/min)		
[Diol]	[BA]	EG-BA	PD-BA	BD-BA
50	25	47.35 ± 0.27	42.29 ± 2.23	48.34 ± 0.85
50	50	40.33 ± 1.64	38.38 ± 2.14	38.74 ± 1.28
100	50	96.90 ± 0.67	89.75 ± 2.52	93.23 ± 1.23

a All values are given in mM/min.

These results give the following rate equation (eq 2) for all reactions:

2 $$\mathrm{Rate}=k_{\mathrm{obs}}\left[\mathrm{diol}\right]$$

The integrated rate equation for this first order reaction is (eq 3):

3 $$\ln{[\mathrm{diol}]}_{t}=-k_{\mathrm{obs}}t+\ln{[\mathrm{diol}]}_{0}$$

The integrated rate equation (eq 3) can be used to determine the rate constant of the reaction (*k*_obs_). That is, for first-order reactions, plotting the ln of the diol concentration as a function of time should yield a linear trend with a slope equal to *k*_obs_. However, because these reactions do not go to completion and free diol remains in solution, the reversibility of the reaction needs to be taken into account when plotting the data (a nonzero [diol]_∞_). The integrated rate equation was, therefore, plotted using ln­([diol] – [diol]_∞_) (Figure 6). Importantly, the best linear fit was found for the ln­([diol] – [diol]_∞_) versus time plot, supporting the previous data. Plots of [diol] – [diol]_∞_ and 1/[diol] – [diol]_∞_ versus time did not result in a linear fit, thus confirming the order of this reaction. Though the initial rates of these reactions were quite similar, the rate constants (*k*_obs_) varied depending on the diol used. Values for *k*_obs_ were 0.057 min^–1^ ± 0.003 for EG-BA, 0.031 min^–1^ ± 0.005 for PD-BA, and 0.124 min^–1^ ± 0.007 for BD-BA. This indicates that the rate-determining step of the formation of PD-BA complexes is significantly slower than those for EG-BA and BD-BA.

**Figure 6:**
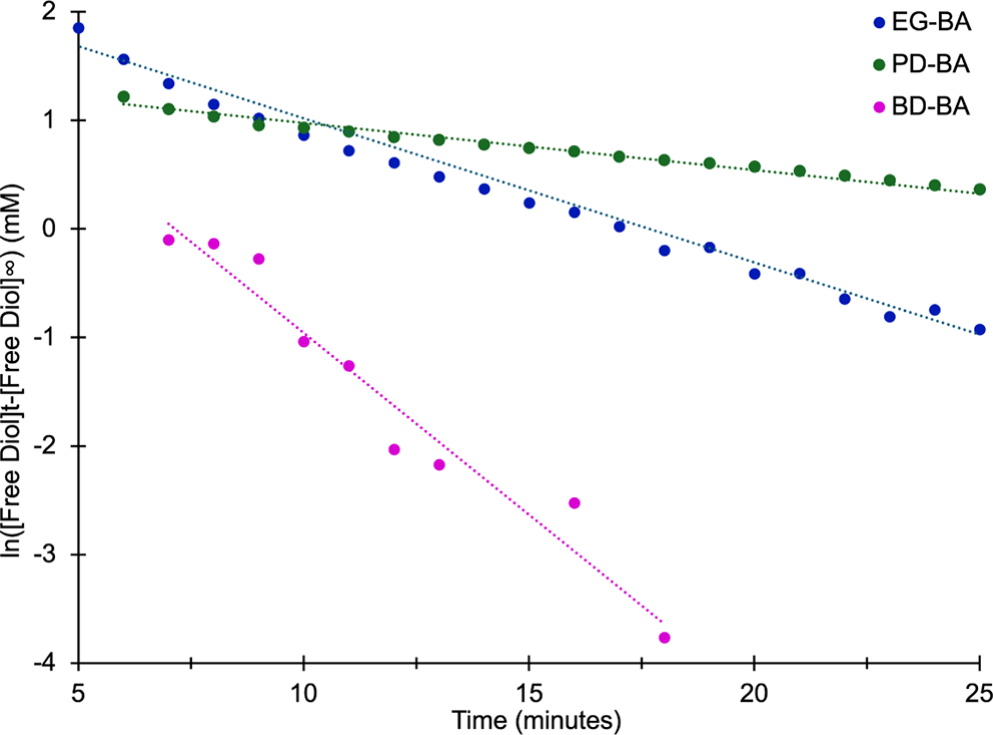
Plot of the integrated rate law, ln­([diol] – [diol]_∞_ versus time for time points of 5 min. Linear lines are shown for EG-BA (blue), PD-BA (green), and BD-BA (fuchsia). Slope values were used to obtain the *k*_obs_ values.

Previous computational reports have proposed various mechanisms for the formation of B–O complexes.^30–36^ All of these report the first step of the mechanism to be the nucleophilic attack on the boron of BA by the oxygen of one of the hydroxyl groups of the diol or polyol to form an open complex before cyclization to the closed diol-BA species. Based on these reports and our initial results, we theorize that the cyclization to form the closed complex is the rate-limiting step. This working theory is supported by the rapid conversion of BD to the open BD-BA complex (indicating that the mechanistic steps needed to form the open complex are fast) and suggests that the size of the closed B–O ring (and therefore the length of the diol) impacts the rate of the reaction. That being said, additional studies are needed to fully understand the energy profile, transition states/activation energies, and the overall mechanism for these reactions.

These combined data suggest that 1,3-cyclic B–O esters are more thermodynamically favored but form at a slower rate than 1,2-cyclic B–O esters. However, these results are inconsistent with our previously published data in which a ∼50:50 mixture of 1,3-Glyc-BA and 1,2-Glyc-BA was observed.^23,28^ To investigate this further, computational experiments were again performed as described above for 1,3-Glyc-BA and 1,2-Glyc-BA. The difference in Δ*G* for the reactions to form 1,3-Glyc-BA and 1,2-Glyc-BA (i.e., ΔΔ*G* = Δ*G*_1,3‑Glyc‑BA_-Δ*G*_1,2‑Glyc‑BA_) was calculated. Interestingly, the ΔΔ*G* values for 1,3-Glyc-BA and 1,2-Glyc-BA were 0.16 kcal/mol in the gas phase and −0.57 kcal/mol when solvated in DMSO. These near-zero values indicate that in the Glyc-BA system there is no thermodynamically favored isomer, which corresponds to our experimental findings of a nearly 50:50 mixture of the two cyclic forms at equilibrium. These findings conflict with our hypothesis from the diol-BA systems that 1,3-cyclic B–O esters are more thermodynamically favored than 1,2-cyclic esters. However, if we structurally compare the two 1,2-cyclic esters, 1,2-Glyc-BA and EG-BA, 1,2-Glyc-BA exhibits an intramolecular hydrogen bond formed between the oxygen of the B–O complex and the free OH of Glyc, which is not available in the closed form of EG-BA (Figure 7). We hypothesize that this interaction stabilizes the 1,2-Glyc-BA complex and thus results in the observed increase in its concentration relative to closed EG-BA. These combined results suggest that both ring stability and intramolecular hydrogen bonds play a role in the overall stability of cyclic polyol-BA complexes.

**Figure 7:**
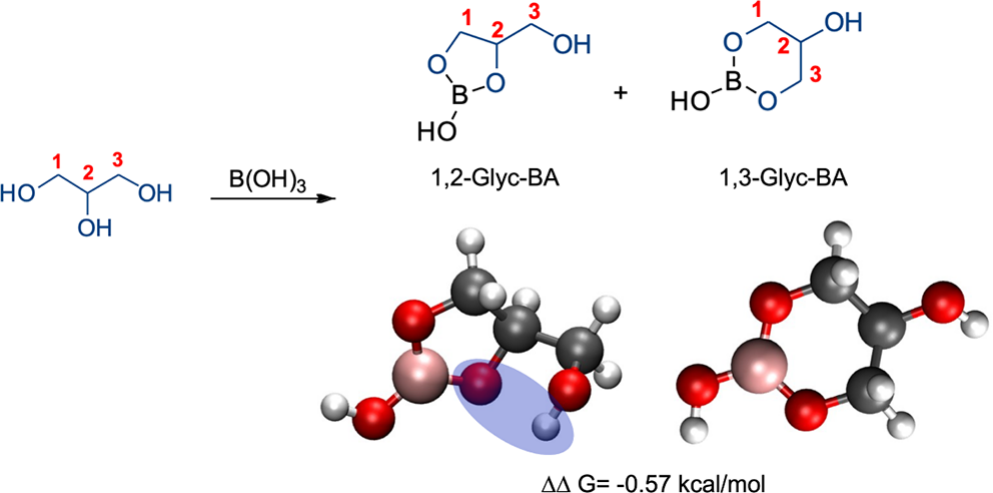
Reaction scheme, computational structural representations, and energy differences between the 1,2-Glyc-BA and 1,3-Glyc-BA complexes. The intramolecular hydrogen bond in 1,2-Glyc-BA is highlighted in blue.

## Conclusions

In conclusion, we investigated the formation of open and closed B–O complexes with EG, PD, BD, and BA in a nonaqueous solvent. The reactions were studied by using NMR spectroscopy to monitor the formation of products. The impact of water content on the conversion to product was also studied, and based on these findings, all further experiments were run at water percentages below 2% to minimize the hydrolysis reaction. PD-BA predominantly forms the closed complex and was the most thermodynamically favored, resulting in ∼76% conversion to product at equilibrium. EG-BA formed both open and closed complexes at a conversion percentage (∼30%) significantly lower than that of PD-BA. Lastly, BD-BA formed only the open complex and had the lowest percent conversion (∼9%). Computational results supported the experimental findings, with the most negative Δ*G* value between the open and closed forms obtained for PD-BA, highlighting its preference for the closed complex. While the Δ*G* value for the open and closed versions of BD-BA was nearly zero, we did not experimentally observe the closed BD-BA complex. We theorize that the open form of BD-BA must be kinetically trapped, not allowing for the formation of the closed complex experimentally. Further kinetic analysis revealed that all reactions are first order with respect to diol. BD-BA has the largest rate constant value, while PD-BA has the smallest. In the context of the closed systems, this would indicate that the rate-limiting step, formation of the cyclic ester, is slower for PD-BA than for EG-BA.

Based on these thermodynamic and kinetic analyses, we theorize that the 1,3-diol-BA species is the thermodynamically preferred closed complex relative to a 1,2- or 1,4-diol-BA system. We also hypothesize that the formation of the closed 1,2-diol-BA complex is kinetically favored and dynamic, leading to a mixture of open and closed 1,2-diol-BA complexes. That being said, to fully understand the energy profiles of diol-BA systems, additional theoretical and spectroscopic experiments will need to be done. Though these findings have provided insights into simple diol systems, computational methods demonstrated that larger polyols have additional interactions that impact the stability of the B–O complexes. Specifically, we found that with Glyc the relative ΔΔ*G* value for the 1,2-Glyc-BA and 1,3-Glyc-BA isomers was −0.57 kcal/mol, suggesting that these complexes are thermodynamically similar in energy, a stark contrast to diol-BA systems. We hypothesize that the improved stabilization in the closed 1,2-Glyc-BA complex relative to EG-BA arises from an additional hydrogen bond between the free OH and the B–O ring. These findings are consistent with our previously reported experimental data in which Glyc formed a ∼50:50 mixture of the 1,2- and 1,3-Glyc-BA complexes. In the future, we will build upon these studies to better understand these stabilizing interactions and explore the impacts of stereochemistry, sterics, and polyol length on complexation.

## Experimental Section

### Materials

1,3-Propanediol (PD), ethylene glycol (EG), 1,4-butanediol (BD), boric acid (BA), and *d*_6_-DMSO were all used as received from Sigma-Aldrich.

### Nuclear Magnetic Resonance Spectroscopy

Nuclear magnetic resonance (^1^H NMR) spectra were obtained using a Bruker Avance DPX-400 NMR spectrometer in *d*_6_-DMSO; chemical shifts are reported in parts per million downfield from tetramethylsilane (δ scale).

Water titrations were performed at 50 mM diol (EG, PD, or BD) and 50 mM BA. Stock solutions of diol and BA (200 mM each) in *d*_6_-DMSO were mixed in equal volumes and diluted with *d*_6_-DMSO and the appropriate volumes of water to obtain the desired solutions (50 mM diol, 50 mM BA, ∼0.75–6%). The actual percentage of water in the samples was calculated using the integration values from DMSO and H_2_O (see SI-Figure S1 for calculation details). Kinetic experiments were run at 50 mM diol and 0.5–2.0 equiv of BA in *d*_6_-DMSO. All experiments were performed by freezing the diol-containing sample prior to BA addition and monitoring the NMR spectrum as a function of time, allowing the sample to thaw in the magnet prior to data collection. Unless otherwise noted, NMR spectra were collected every minute for the first hour and then every hour for as long as 7 days until no changes were observed in the spectrum, at which time the system was deemed to have reached equilibrium.

### Computational Methods

Density Functional Theory (DFT) calculations were run using the Gaussian16 suite of programs.^37^ The SMD implicit solvation model^38^ was used to simulate the experimental environment, i.e., in DMSO. All optimization and frequency calculations were at M06-2X/311+G­(2d,p) level of theory (in DMSO), the default (UltraFine) grid was used, and thermodynamic properties were calculated at room temperature (298.15 K). The utilities within the MStor program^29^ were used to construct all rotational conformers of the diol-boric acid complexes. Initial geometries were generated by identifying all bonds that allow rotations (e.g., C–C single bonds) and the possible number of unique rotations (e.g., three, 60°, 180°, 300°—for rotations around sp^3^ hybridized carbons). An initial geometry was created for each possible rotamer, e.g., BD-BA has 5 rotatable bonds, each with three possible rotations, which results in 125 unique dihedral combinations. These structures were then optimized and analyzed for “uniqueness.” Geometries are considered unique if they are not mirror images and if their dihedrals differ by more than 20°. The final number of unique, stable (e.g., nontransition state) structures for each boron ester is shown in Table 1. Finally, the multiconfigurational local harmonic approximation^39^ within the MStor program^29^ was used to account for all the rotamers and their mirror images in the Gibb’s free energy calculations.

## Supplementary Material





## Notes

Additional full water analysis and calculations; NMR spectra; and full kinetic plots (PDF)
Geometries of the optimized, unique rotamers; geometries and relative energies (ZIP)

## Abbreviations

BA: boric acid
DMSO: dimethyl sulfoxide
EG: ethylene glycol
PD: 1,3-propanediol
BD: 1,4-butanediol
NMR: nuclear magnetic resonance
DFT: Density Functional Theory
Glyc: glycerol
Ery: erythritol

## References

[1] Hahn J., Monakhova Y. B., Hengen J., Kohl-Himmelseher M., Schössler J., Hahn H., Kuballa T., Lachenmeier D. W. (2014). “Electronic
Cigarettes: Overview of Chemical Composition and Exposure Estimation”. Tob. Induc. Dis..

[2] Song H., Jin R., Kang M., Chen J. (2013). “Progress in Synthesis of
Ethylene Glycol through C1 Chemical Industry Routes”. Cuihua xuebao/Chin. J. Catal..

[3] Haas T., Jaeger B., Weber R., Mitchell S. F., King C. F. (2005). “New
Diol Processes: 1,3-Propanediol and 1,4-Butanediol”. Appl. Catal., A.

[4] De
Souza F. M., Kahol P. K., Gupta R. K. (2021). “Introduction
to Polyurethane Chemistry”. ACS Symp.
Ser..

[5] Muñiz, M. K. “The Pinacol Rearrangement”. In Comprehensive Organic Synthesis: second ed.; Elsevier Ltd, 2014, Vol. 3, pp. 741–756.

[6] Zhao Z., Yao X., Zhang Z., Chen L., He C., Chen X. (2014). “Boronic
Acid Shell-Crosslinked Dextran-b-PLA Micelles for Acid-Responsive
Drug Delivery”. Macromol. Biosci..

[7] Yang H., Zhang C., Li C., Liu Y., An Y., Ma R., Shi L. (2015). “Glucose-Responsive
Polymer Vesicles Templated
by α-CD/PEG Inclusion Complex”. Biomacromolecules.

[8] Cash J. J., Kubo T., Bapat A. P., Sumerlin B. S. (2015). “Room-Temperature
Self-Healing Polymers Based on Dynamic-Covalent Boronic Esters”. Macromolecules.

[9] Cash J. J., Kubo T., Dobbins D. J., Sumerlin B. S. (2018). “Maximizing
the Symbiosis of Static and Dynamic Bonds in Self-Healing Boronic
Ester Networks”. Polym. Chem..

[10] Scott H. R., Davis A. N., Peters G. M. (2023). “Cooperative
Crosslinking
in Polyvinyl Alcohol Organogels”. Soft
Matter.

[11] Peters G.
M., Chi X., Brockman C., Sessler J. L. (2018). “Polyvinyl Alcohol-Boronate
Gel for Sodium Hydroxide Extraction”. Chem. Commun..

[12] Deng C. C., Brooks W. L. A., Abboud K. A., Sumerlin B. S. (2015). “Boronic
Acid-Based Hydrogels Undergo Self-Healing at Neutral and Acidic PH”. ACS Macro Lett..

[13] Pettignano A., Grijalvo S., Häring M., Eritja R., Tanchoux N., Quignard F., Díaz
Díaz D. (2017). “Boronic Acid-Modified
Alginate Enables Direct Formation of Injectable, Self-Healing and
Multistimuli-Responsive Hydrogels”. Chem.
Commun..

[14] Dong Y., Wang W., Veiseh O., Appel E. A., Xue K., Webber M. J., Tang B. C., Yang X. W., Weir G. C., Langer R. (2016). “Injectable
and Glucose-Responsive Hydrogels
Based on Boronic Acid-Glucose Complexation”. Langmuir.

[15] Peters G. M., Skala L., Plank T., Hyman B., Manjunatha
Reddy G. N., Marsh A., Brown S., Davis J. (2014). “A
G_4_·K^+^ Hydrogel Stabilized by an Anion”. J. Am. Chem. Soc..

[16] Peters G. M., Skala L., Plank T., Oh H., Manjunatha
Reddy G. N., Marsh A., Brown S., Raghavan S., Davis J. (2015). “G4-Quartet·M^+^ Borate Hydrogels”. J. Am. Chem. Soc..

[17] Peters G. M., Skala L. P., Davis J. T. (2016). “A Molecular Chaperone for
G_4_-Quartet Hydrogels”. J.
Am. Chem. Soc..

[18] Casassa E. Z., Sarquis A. M., Van Dyke C. H. (1986). “The Gelation of Polyvinyl
Alcohol with Borax”. J. Chem. Educ..

[19] Van
Duin M., Peters J. A., Kieboom A. P. G., Van Bekkum H. (1984). “Studies
on Borate Esters 1: The PH Dependence of the Stability of Esters of
Boric Acid and Borate in Aqueous Medium as Studied by ^11^B NMR”. Tetrahedron.

[20] Benner K., Klüfers P. (2000). “A
Combined X-Ray and NMR Study of Borate Esters
of Furanoidic Cis-1,2-Diols”. Carbohydr.
Res..

[21] Sinton S. (1987). “Complexation
Chemistry of Sodium Borate with Poly­(Vinyl Alcohol) and Small Diols.
A ^11^B NMR Study”. Macromolecules.

[22] Peters G. M., Davis J. T. (2014). “Controlling the Transmembrane
Transport of
Nucleosides”. Supramol. Chem..

[23] Scott H. R., Pearson C. J., Ealley L. C., Boardman B. M., Peters G. M. (2024). “Tuning
Glycerol Plasticization of Chitosan with Boric Acid”. Int. J. Biol. Macromol..

[24] Daniels E. L., Runge J. R., Oshinowo M., Leese H. S., Buchard A. (2023). “Cross-Linking
of Sugar-Derived Polyethers and Boronic Acids for Renewable, Self-Healing,
and Single-Ion Conducting Organogel Polymer Electrolytes”. ACS Appl. Energy Mater..

[25] Angelova L. V., Leskes M., Berrie B. H., Weiss R. G. (2015). “Selective
Formation of Organo, Organo-Aqueous, and Hydro Gel-like Materials
from Partially Hydrolysed Poly­(Vinyl Acetate)­s Based on Different
Boron-Containing Crosslinkers”. Soft
Matter..

[26] Bachelier N., Verchere J.-F. (1995). “Formation
of Neutral Complexes of Boric Acid
with 1,3-Diols in Organic Solvents and in Aqueous Solution”. Polyhedron.

[27] Fortuny A., Coll M. T., Kedari C. S., Sastre A. M. (2014). “Effect
of
Phase Modifiers on Boron Removal by Solvent Extraction Using 1,3 Diolic
Compounds”. J. Chem. Technol. Biotechnol..

[28] Coer O. E., Davidson B. L., Boardman B. M., Peters G. M. (2025). “Modulating
Thermal Stability and Flexibility in Chitosan Films with Neutral Polyol-Boric
Acid Complexes”. Biomacromolecules.

[29] Chen W., Zheng J., Bao J. L., Truhlar D. G., Xu X. (2023). “MSTor
2023: A New Version of the Computer Code for Multistructural Torsional
Anharmonicity, Now with Automatic Torsional Identification Using Redundant
Internal Coordinates”. Comput. Phys.
Commun..

[30] Bharadwaj V. S., Crowley M. F., Peña M. J., Urbanowicz B., O’Neill M. (2020). “Mechanism and Reaction Energy
Landscape for
Apiose Cross-Linking by Boric Acid in Rhamnogalacturonan II”. J. Phys. Chem. B.

[31] Kunimoto M., Bothe D., Tamura R., Oyanagi T., Fukunaka Y., Nakai H., Homma T. (2018). “Spectroscopic
and Computational
Analyses of Liquid-Liquid Interfacial Reaction Mechanism of Boric
Acid Esterification with 2,2,4-Trimethyl-1,3-Pentanediol in Boron
Extraction Processes”. J. Phys. Chem.
C.

[32] Bacchiocchi C., Petroselli M. (2024). “Elucidation of the Mechanism of the Esterification
of Boric Acid with Aliphatic Diols: A Computational Study to Help
Set the Record Straight”. Chem. Commun..

[33] Bhat K. L., Hayik S., Bock C. W. (2003). “A
Computational Study of
the Formation of a Boron–Oxygen–Carbon Linkage. The
Reaction of Monohydroxy Borane with Methanol”. J. Mol. Struct.: THEOCHEM.

[34] Bhat K. L., Hayik S., Corvo J. N., Marycz D. M., Bock C. W. (2004). “A
Computational Study of the Formation of 1,3,2-Dioxaborolane from the
Reaction of Dihydroxy Borane with 1,2-Ethanediol”. J. Mol. Struct.: THEOCHEM.

[35] Monajemi H., Cheah M. H., Lee V. S., Zain S. M., Tajuddin
Wan Abdullah W. A. (2014). “On the Kinetics and Reaction Mechanisms of
Boronic Acid in Interaction with Diols for Non-Enzymatic Glucose Monitoring
Applications: A Hybrid DFT Study”. RSC
Adv..

[36] Li H., Li H., Dai Q., Li H., Brédas J. -L. (2018). “Hydrolytic
Stability of Boronate Ester-Linked Covalent Organic Frameworks”. Adv. Theory Simul.

[37] Frisch, M. J. ; Trucks, G. W. ; Schlegel, H. B. ; Scuseria, G. E. ; Robb, M. A. ; Cheeseman, J. R. ; Scalmani, G. ; Barone, V. ; Petersson, G. A. ; Nakatsuji, H. , “Gaussian 16, Revision C.01”. Gaussian, Inc.: Wallingford, CT, 2016.

[38] Marenich A. V., Cramer C. J., Truhlar D. G. (2009). “Universal
Solvation Model
Based on Solute Electron Density and on a Continuum Model of the Solvent
Defined by the Bulk Dielectric Constant and Atomic Surface Tensions”. J. Phys. Chem. B.

[39] Zheng J., Yu T., Papajak E., Alecu I. M., Mielke S. L., Truhlar D. G. (2011). “Practical
Methods for Including Torsional Anharmonicity in Thermochemical Calculations
on Complex Molecules: The Internal-Coordinate Multi-Structural Approximation”. Phys. Chem. Chem. Phys..

