# Supplementary material for: Kinetic and Thermodynamic Investigation into the Formation of Diol-Boric Acid Complexes Using NMR and Computational Analysis
Source: ACS Omega. 2025 Nov 17;10(46):55824–31. doi: 10.1021/acsomega.5c07314 (PMC12658599; doi:10.1021/acsomega.5c07314)
Supplement: Supplementary file 1 [file ao5c07314_si_001.pdf]

Supporting Information for

Kinetic and thermodynamic investigation into the formation of diol-boric acid complexes using NMR and computational analysis

Brandy L. Davidson, Elaina X. Manyin, Nathan E. Morris, Isaiah Sumner,* Brycelyn M. Boardman,* and Gretchen M. Peters*

Department of Chemistry and Biochemistry, James Madison University, Harrisonburg, Virginia 22807, United States

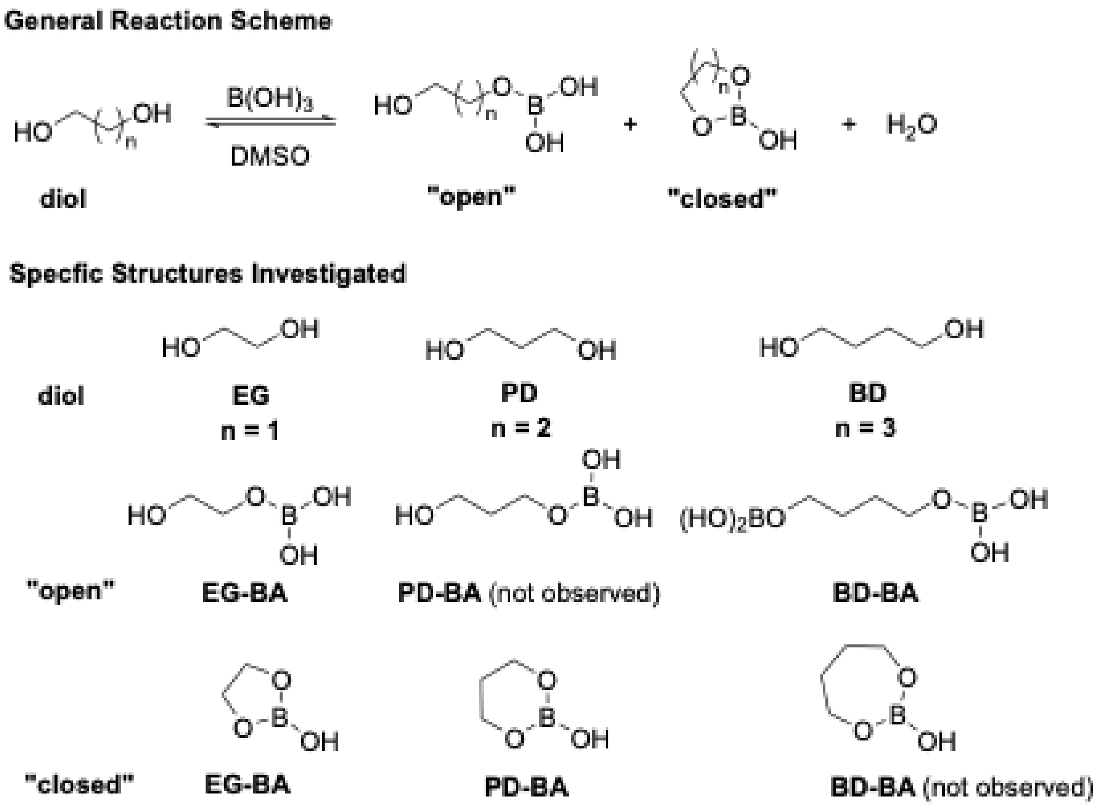

Figure S0. (Top) General reaction scheme for the formation of open and closed diol-BA complexes. (Bottom) Specific structures investigated including diols (EG, PD, and BD) and open and closed structures of diol-BA complexes (EG-BA, PD-BA, and BD-BA). It should be noted that while the structure of open PD-BA and closed BD-BA are shown, they were not experimentally observed.

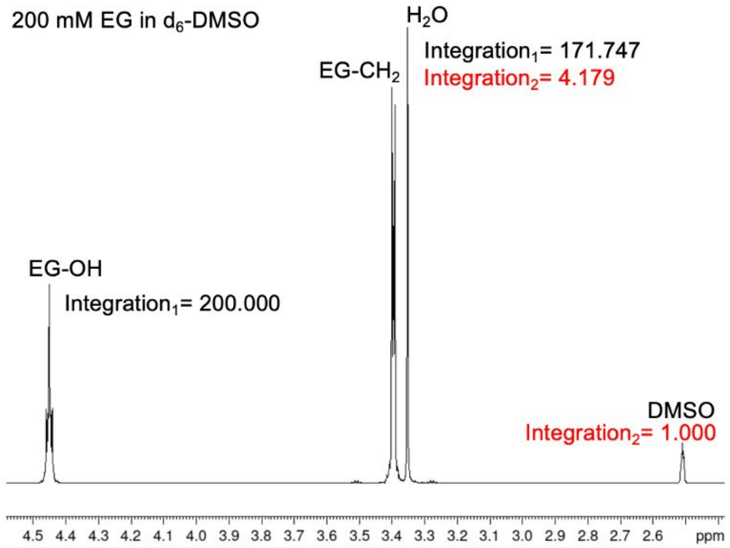

1). Solve for % water in standard sample

Procedure: given 200 mM EG sample in 0.5 mL of d6-DMSO

- Set integration$_1$ of EG-OH to [sample]
- Resulting integration$_1$ of H_2O=[H_2O] in the sample
- Convert [H_2O] to mL of H_2O
- Divide mL of H_2O by total solution volume

Ex: Integration$_1$ of EG-OH=200.00, Integration$_1$ of H_2O= 171.747

$$\frac{0.171747\ mols}{L} \text{ x } \frac{0.0005L}{1} \text{ x } \frac{18g}{1\ mol} \text{ x } \frac{1g}{1\ mL} = 0.001545 \text{ mL } H_2O$$

$$\frac{0.001545\ mL\ H_2O}{0.5\ mL\ Solution} \text{ x } 100 = 0.309\ \%\ H_2O \text{ in solution}$$

2). Solve for integration to water % conversion factor

Procedure:

- Set integration$_2$ of DMSO= 1.000 and record integration$_2$ of H_2O
- Use known % water in sample to set up conversion factor for Integration of H_2O_{expt}

Ex: Integration$_2$ of DMSO=1.000, Integration$_2$ of H_2O= 4.179

$$\frac{4.179}{0.309} = \frac{Integration\ H_2O_{expt}}{x\ \%\ H_2O\ in\ sample}$$

$$x\ \%\ H_2O \text{ in sample} = \text{Integration of } H2O_{expt} \text{ x } 0.73$$

*Conversion factor was calculated for 200 mM PD, and 200 mM BD to get an average conversion factor of 0.069 $\pm$0.004. This average conversion factor was then used to determine the % water in every NMR sample.

Figure S1.(Top) ^{1}H NMR spectrum of 200 mM EG in d6-DMSO with peaks and integrations labeled. (Bottom) Sample calculation for finding the integration to percent water conversion factor. An integration to percent water conversion factor was found by performing the detailed calculation on 200 mM PD and BD in addition to the EG shown in the example.

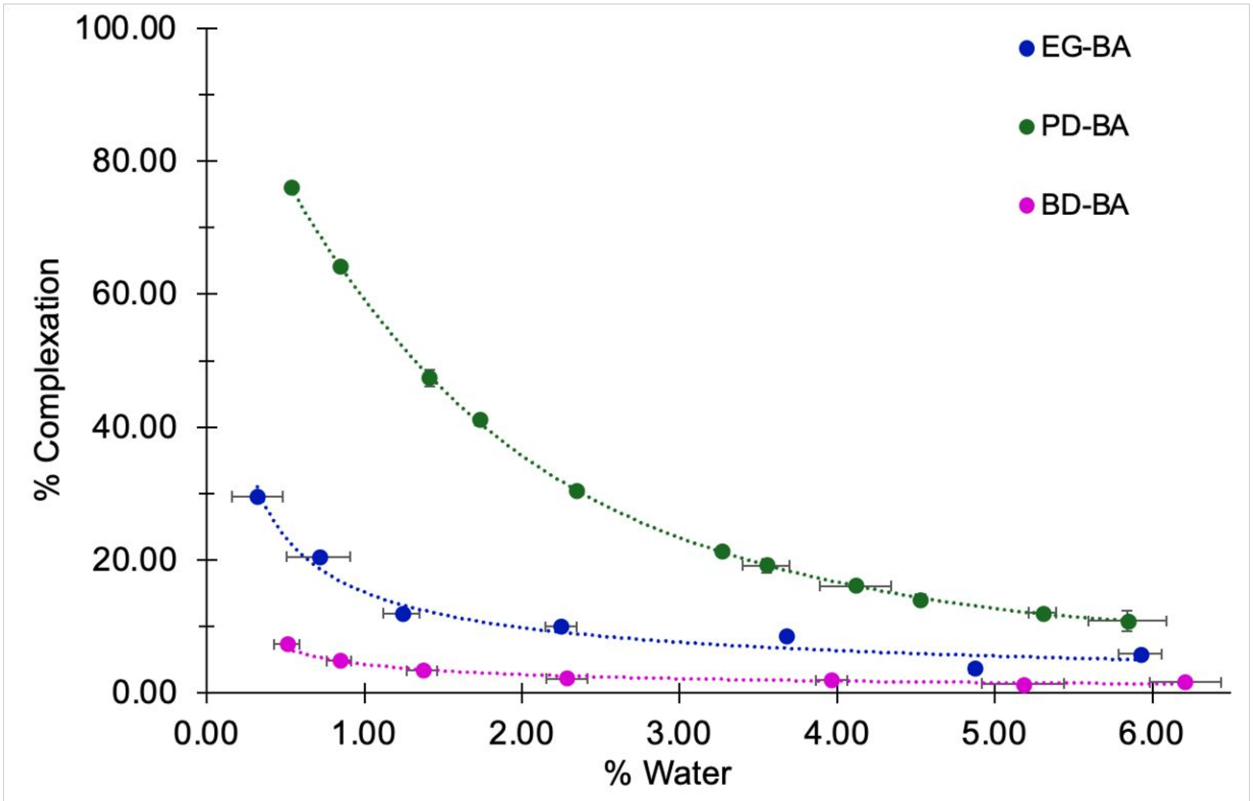

Figure S2. Plot of percent total complexation versus percent water for 50 mM diol and 50 mM BA. All solutions were allowed to reach equilibrium before percent complexation was recorded. EG-BA (blue), PD-BA (green), and BD-BA (fuchsia).

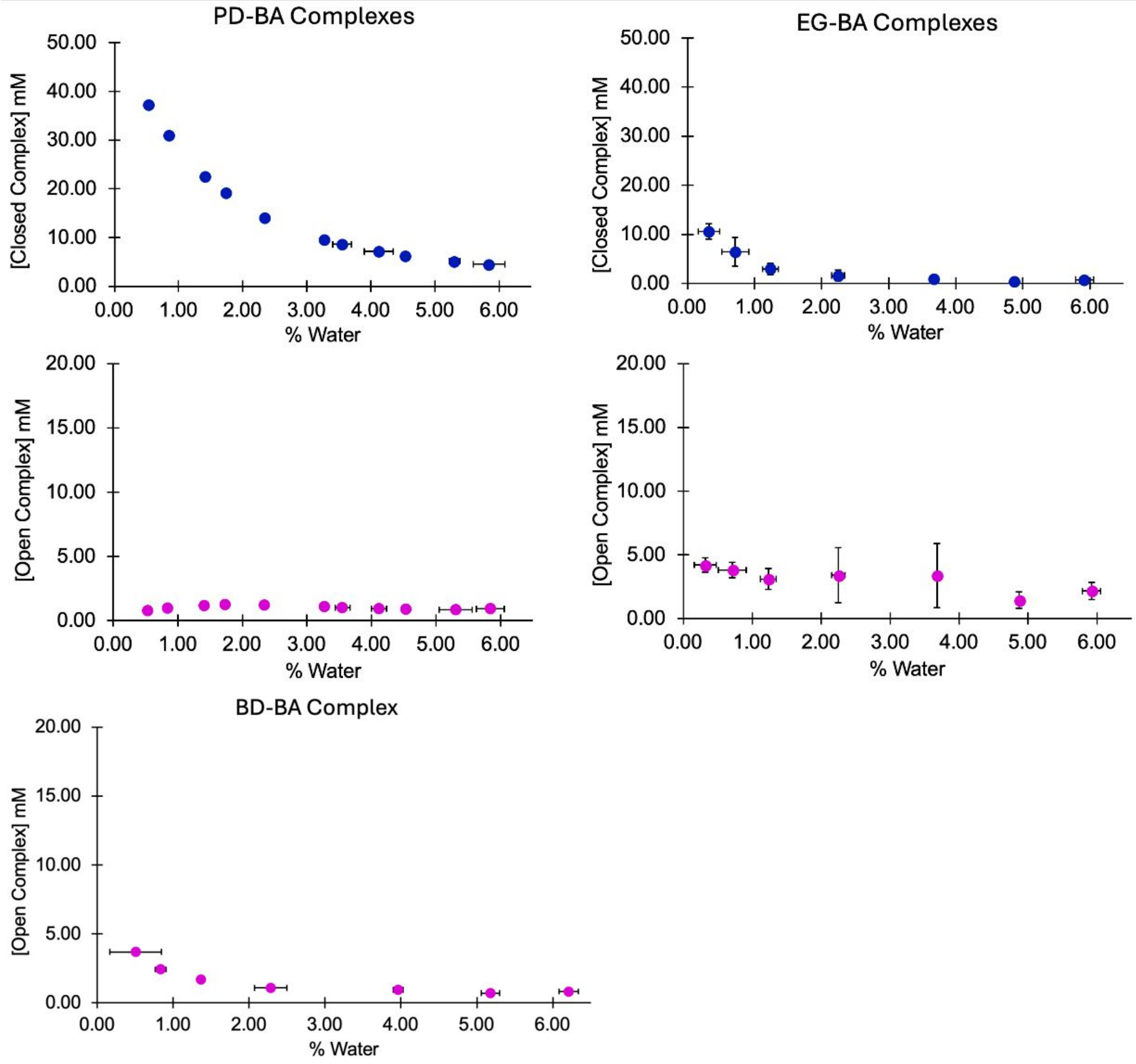

Figure S3. Plots of closed (blue) and (or) open (fuchsia) complex(es) concentration versus percent water content for EG-BA, PD-BA, and BD-BA.

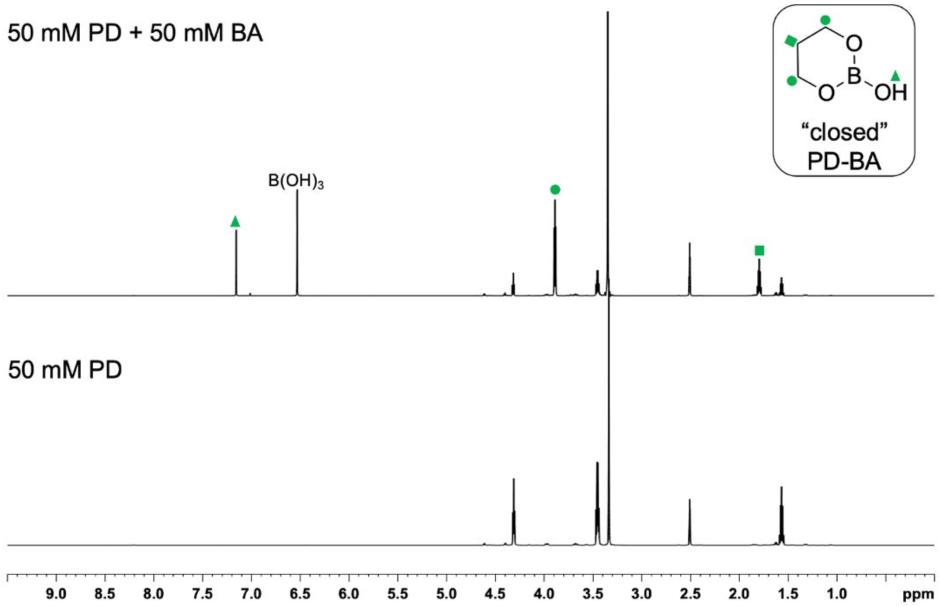

Figure S4. ^{1}H NMR of 50 mM PD (bottom) and 50mM PD + 50 mM BA (top) in d6-DMSO after the reaction has reached equilibrium. Only the closed complex and free diol remain when the reaction reaches equilibrium.

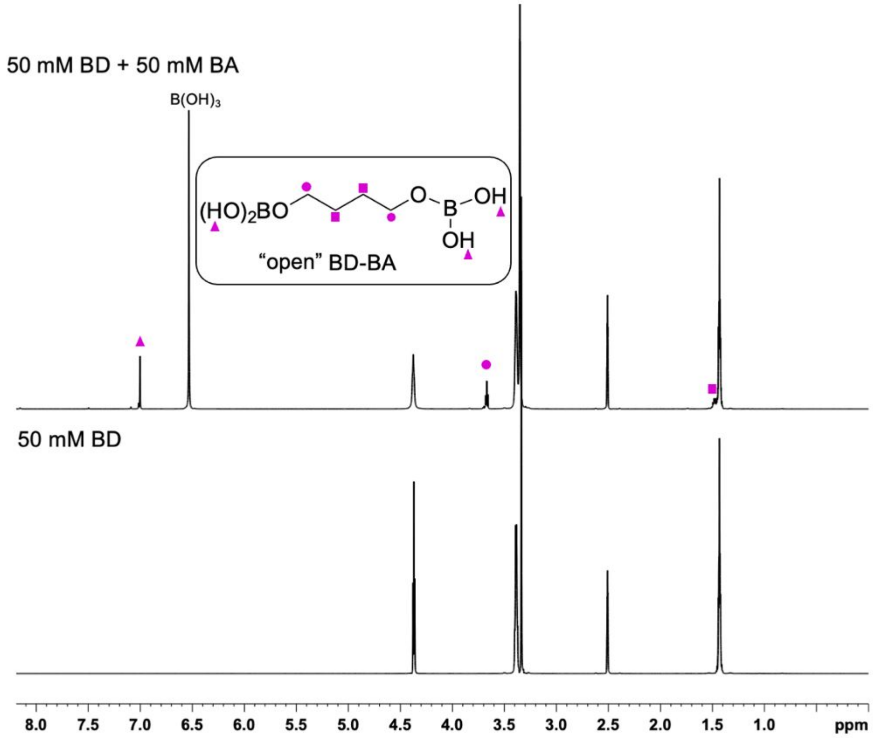

Figure S5. ^{1}H NMR of 50 mM BD (bottom) and 50mM BD + 50 mM BA (top) in d6-DMSO after the reaction has reached equilibrium. Only the open complex and free diol remain when the reaction reaches equilibrium.

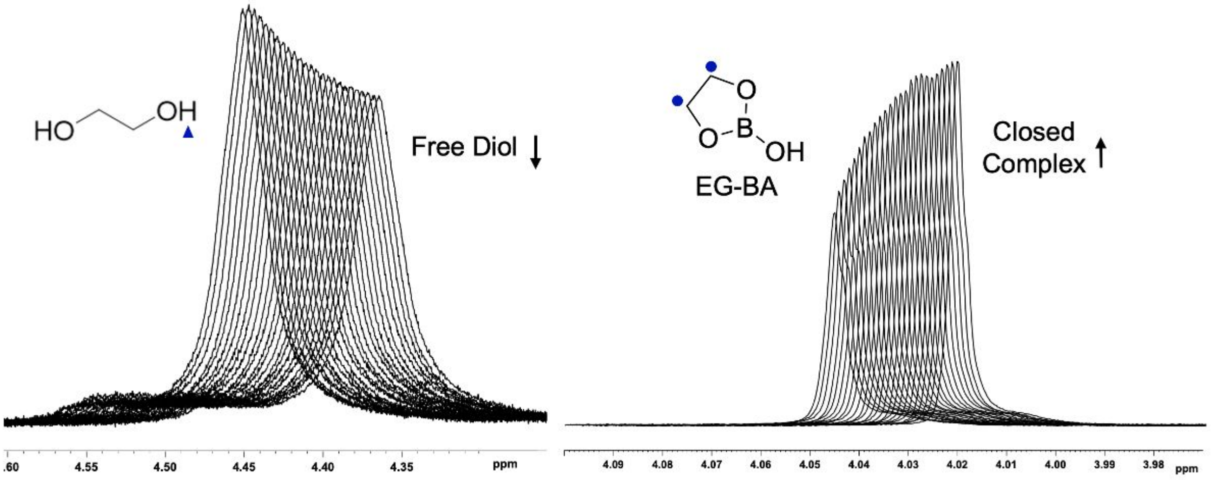

Figure S6. Stacked 1D ^{1}H NMR of 50 mM EG and 50 mM BA at increasing reaction times. The OH signal of free EG decreases as the CH_2 for EG-BA increases as the reaction progresses.

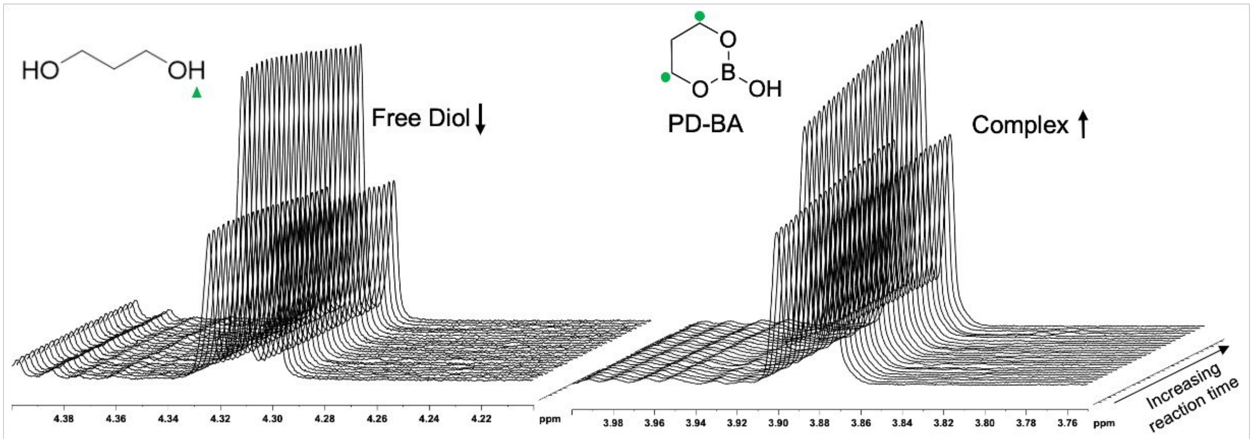

Figure S7. Stacked 1D ^{1}H NMR of 50 mM PD and 50 mM BA at increasing reaction times. The OH signal of free PD decreases as the CH_2 for PD-BA increases as the reaction progresses.

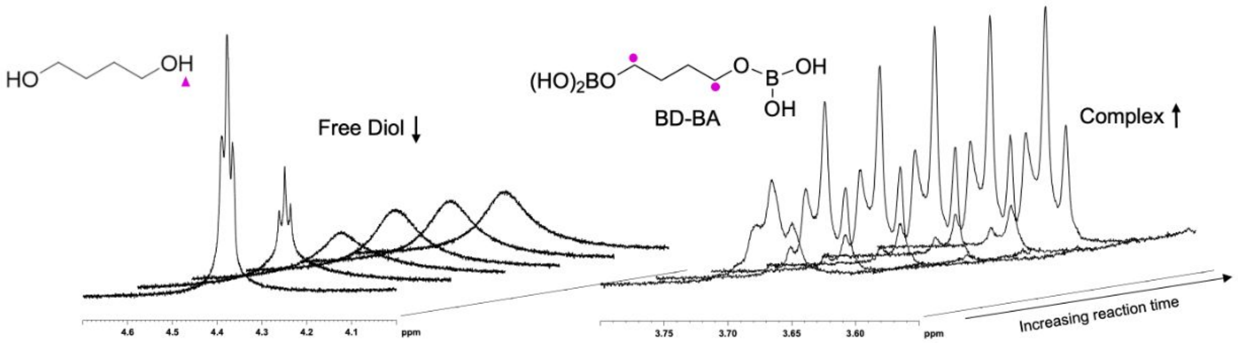

Figure S8. Stacked 1D ^{1}H NMR of 50 mM BD and 50 mM BA at increasing reaction times. The OH signal of free BD decreases as the CH_2 for BD-BA increases as the reaction progresses.

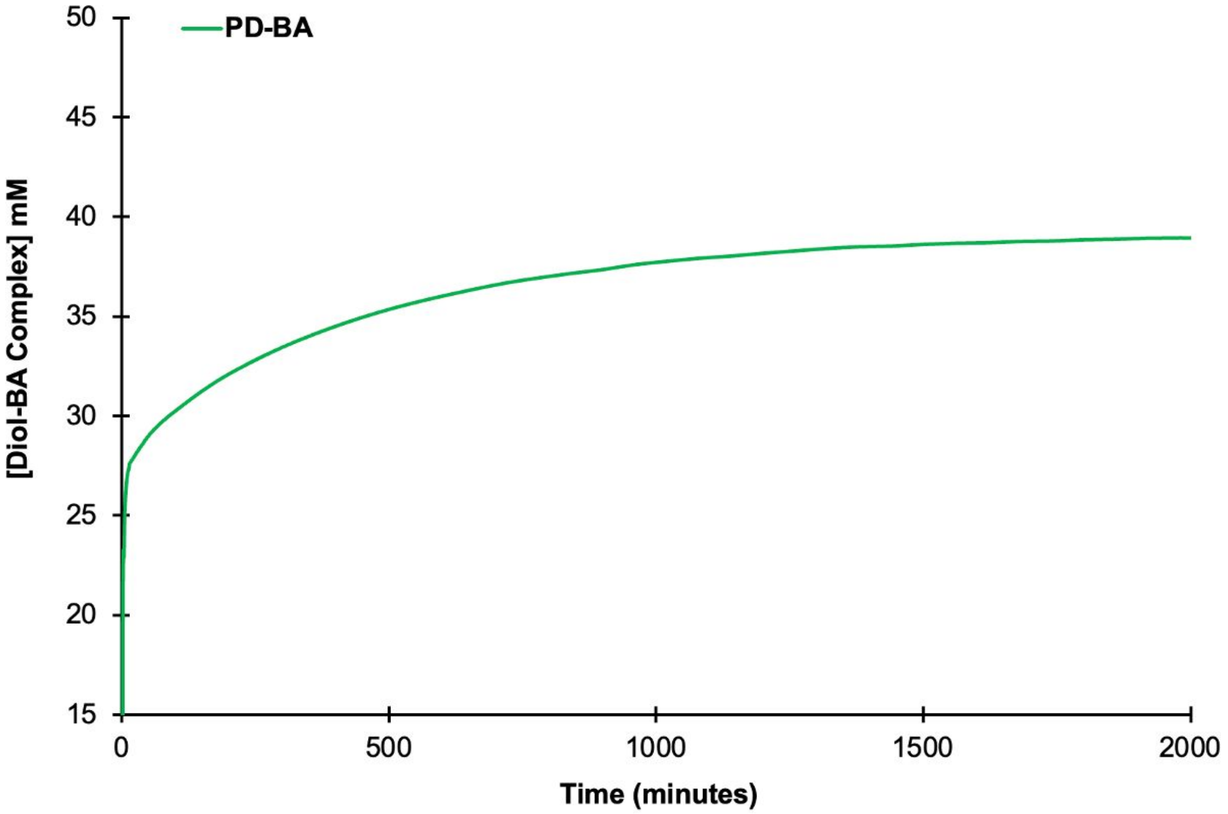

Figure S9. Full concentration versus time plot for PD-BA. Concentration values calculated from NMR experiments.

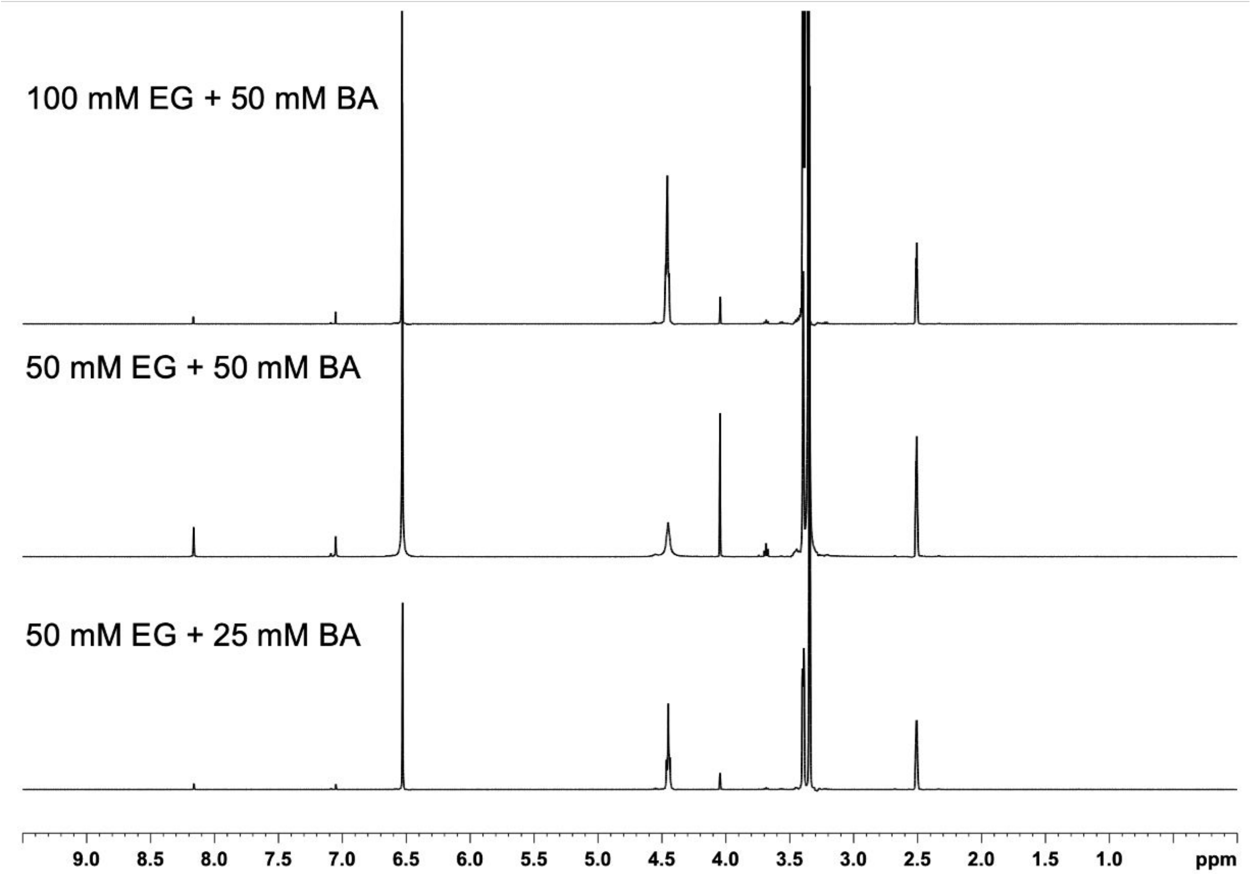

Figure S10. Full ^{1}H NMR spectra of EG and BA concentration ratios used in kinetic experiments. All spectra shown were at the 10 minute time point in d6-DMSO.

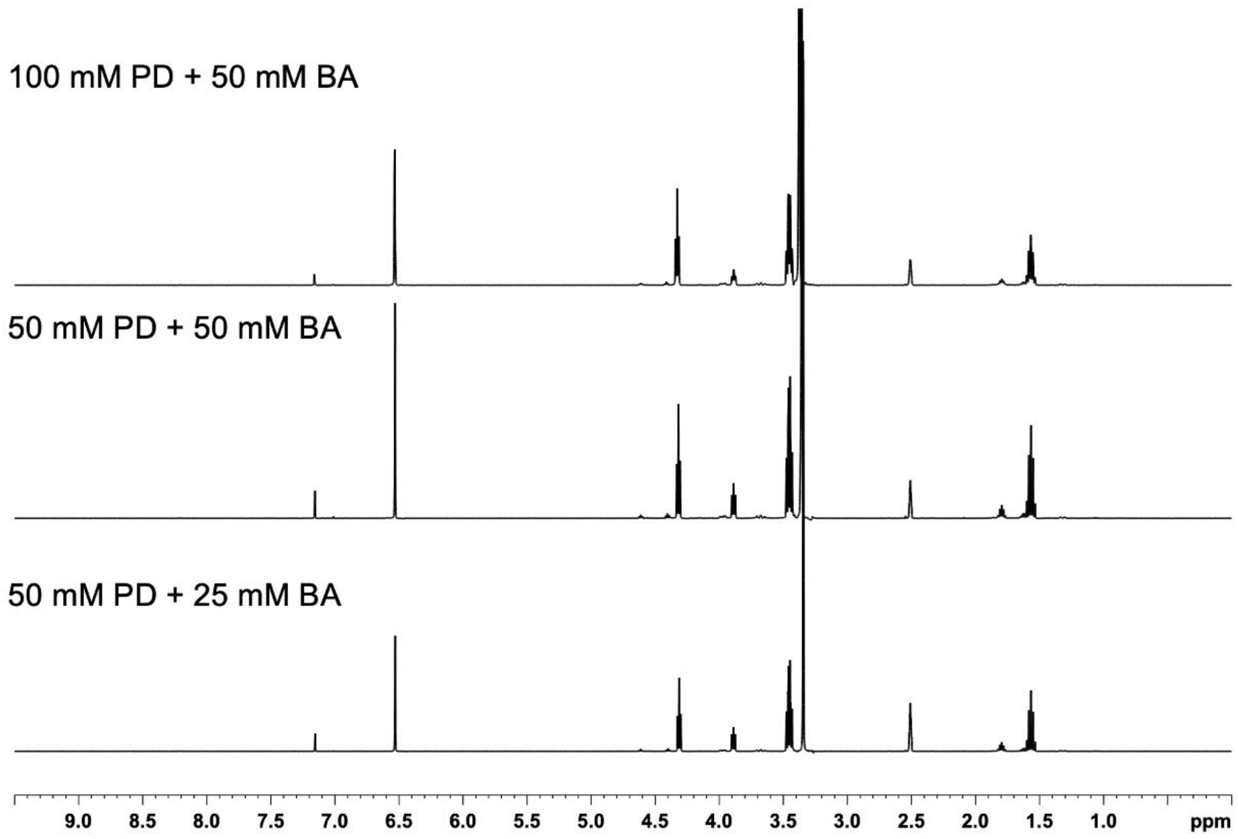

Figure S11. Full ^{1}H NMR spectra of PD and BA concentration ratios used in kinetic experiments. All spectra shown were at the 10 minute time point in d6-DMSO.

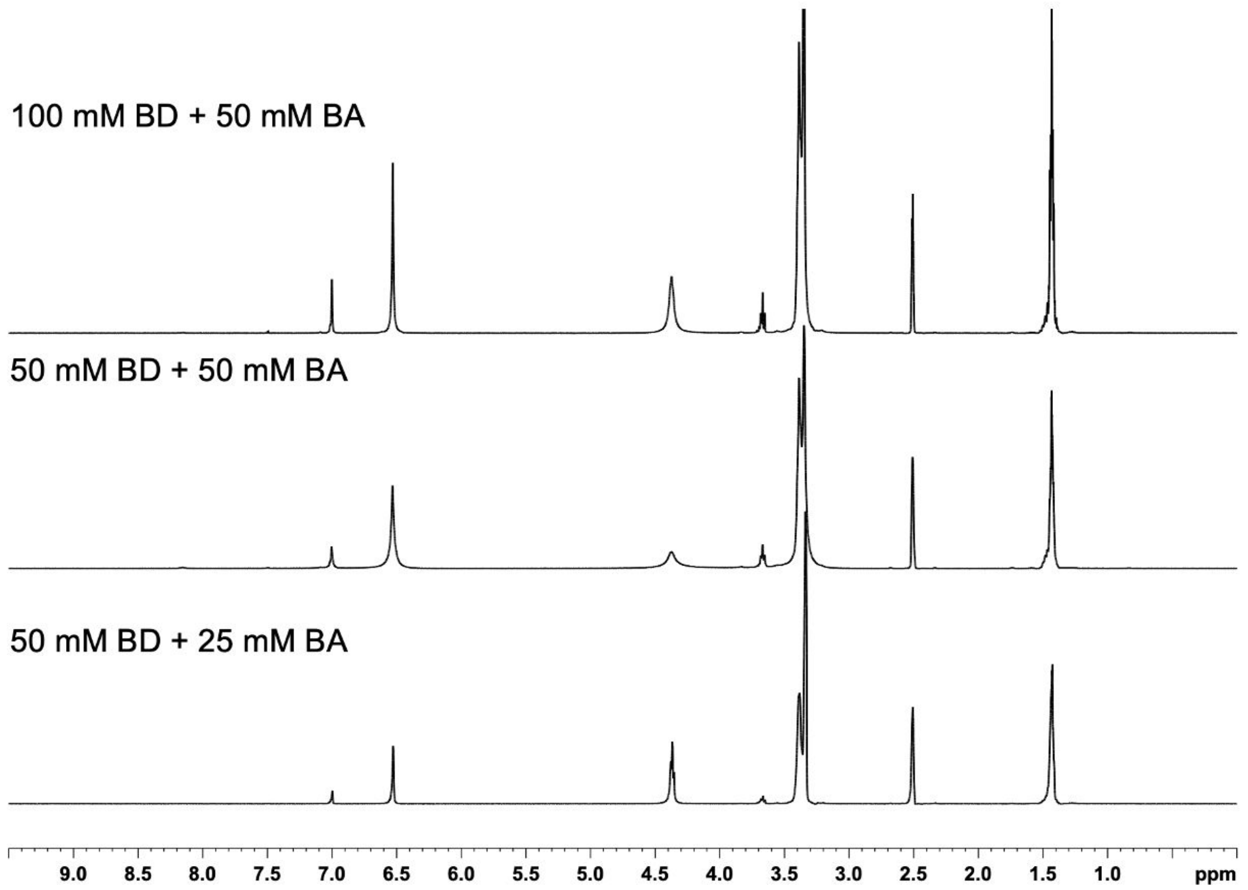

Figure S12. Full ^{1}H NMR spectra of BD and BA concentration ratios used in kinetic experiments. All spectra shown were at the 10 minute time point in d6-DMSO.

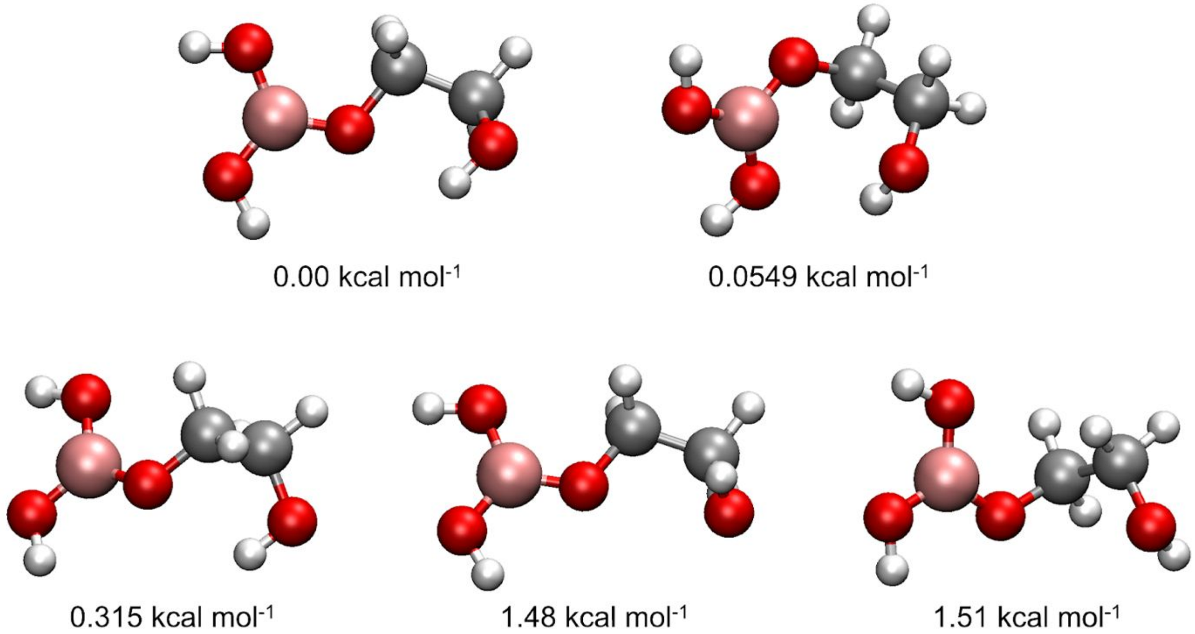

Figure S13. Five lowest energy EG-BA rotamers (excluding mirror images). The relative electronic energies are shown in kcal mol^{-1}.

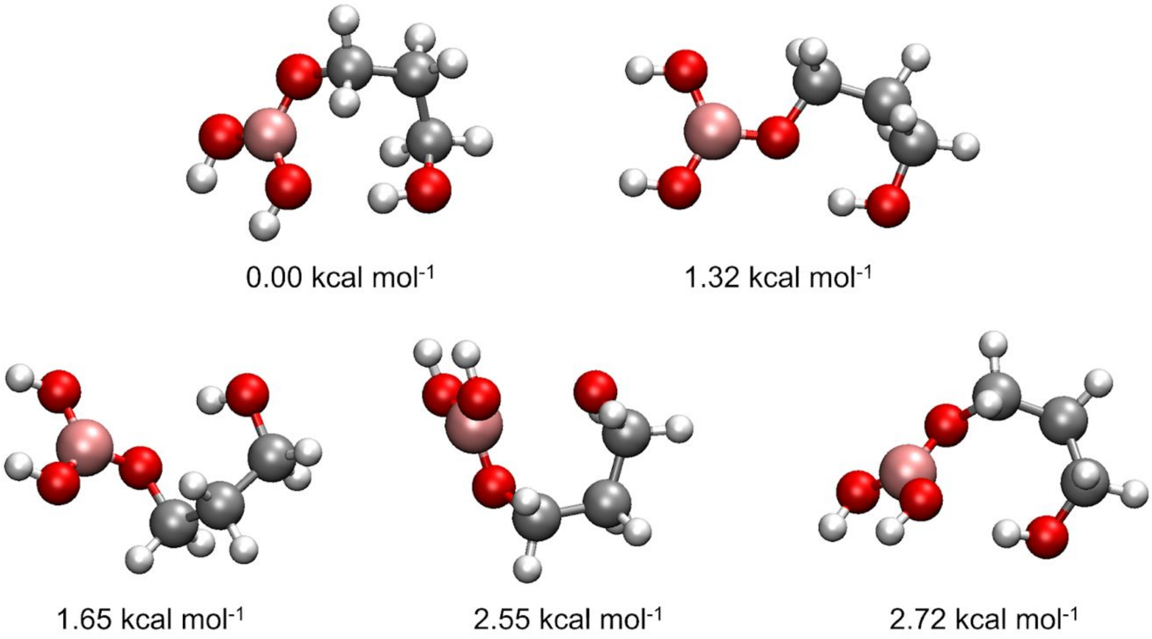

Figure S14. Five lowest energy PD-BA rotamers (excluding mirror images). The relative electronic energies are shown in kcal mol^{-1}.

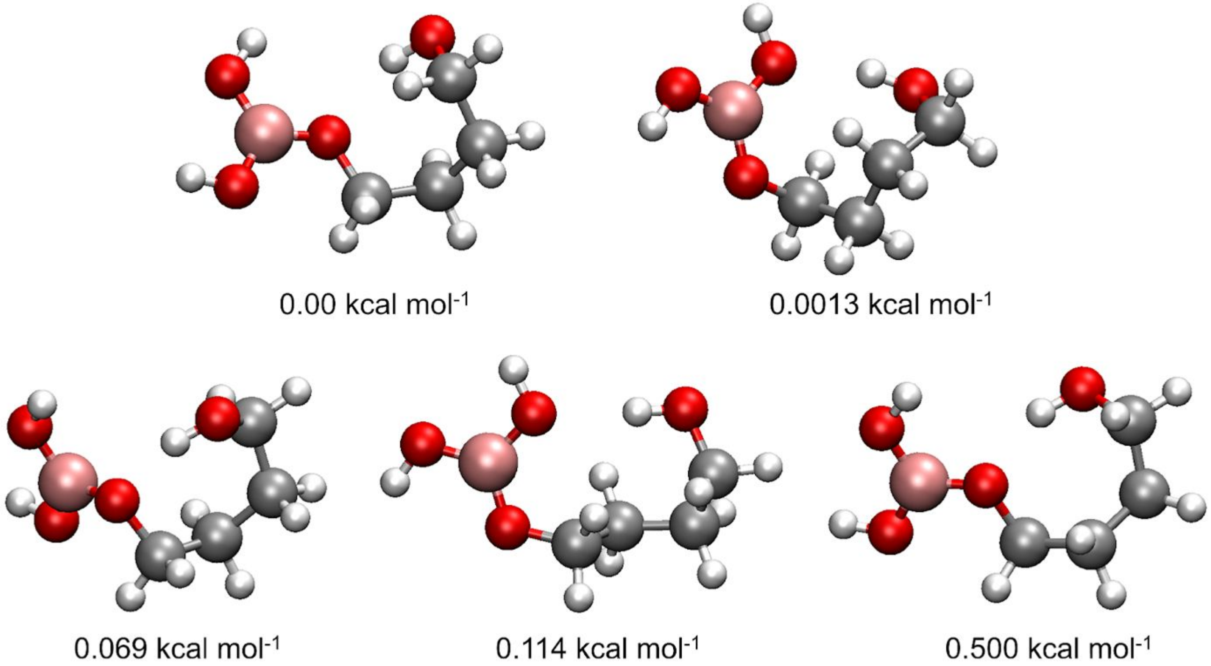

Figure S15. Five lowest energy BD-BA rotamers (excluding mirror images). The relative electronic energies are shown in kcal mol^{-1}.
